# Technical aspects of transpapillary biopsy for gallbladder cancer using a novel cholangioscope

**DOI:** 10.1055/a-2173-7893

**Published:** 2023-09-27

**Authors:** Takeshi Ogura, Kimi Bessho, Nobuhiro Hattori, Mitsuki Tomita, Hiroki Nishikawa

**Affiliations:** 1 Endoscopy Center, Osaka Medical and Pharmaceutical University Hospital, Osaka, Japan; 2 Second Department of Internal Medicine, Osaka Medical and Pharmaceutical University Hospital, Osaka, Japan

Cytology of bile juice obtained under endoscopic retrograde cholangiopancreatography (ERCP) guidance is the gold standard technique for obtaining histopathological evidence of gallbladder cancer. However, the diagnostic yield of this technique is insufficient. Endoscopic ultrasound-guided fine-needle aspiration (EUS-FNA) has recently been performed for gallbladder lesions. However, because the gallbladder has a lumen, inadequate EUS-FNA can lead to bile leakage or cancer cell dissemination 1 2 . Therefore, it is sometimes difficult to obtain histological tissue samples from gallbladder tumors. Although transpapillary gallbladder tumor biopsy under cholangioscopy guidance may be useful, the insertion of a cholangioscope into the gallbladder through the cystic duct is challenging. The recent introduction of a novel tapered cholangioscope (eyeMAX, Micro-Tech, China) may address these challenges ( Fig. 1 ). The tip of this scope is significantly tapered, facilitating its smooth insertion into the target site. In addition, the scope features a working channel of 1.8 mm and a dedicated biopsy forceps, with a cup length of 1.6 mm, which enables the retrieval of large quantities of histological tissue. Herein we describe the technical aspects of the use of this scope for performing transpapillary biopsy of gallbladder cancer.

**Fig. 1:**
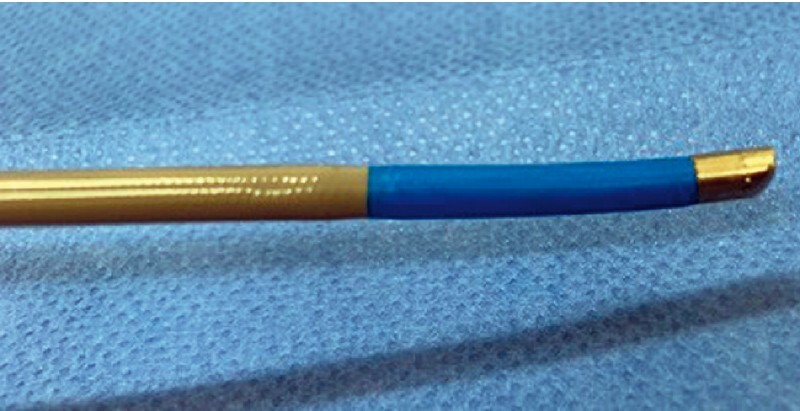
Novel cholangioscope (eyeMAX, Micro-Tech, China) with an extremely tapered tip.

First, following successful biliary cannulation, an ERCP catheter was inserted into the cystic duct. Then, contrast medium was injected and cholangiography was performed, revealing a filling defect with the appearance of a gallbladder tumor ( Fig. 2 ). Then, a guidewire was successfully inserted in the gallbladder, followed by the insertion of the novel cholangioscope into the gallbladder ( Fig. 3 ). Once the gallbladder tumor was visualized ( Fig. 4 ), forceps biopsy was performed without any adverse events ( Fig. 5 , Video 1 ). Histological analysis of the biopsy specimen identified the mass as adenocarcinoma. Following surgical resection, the patient’s diagnosis was confirmed as gallbladder cancer.

**Fig. 2:**
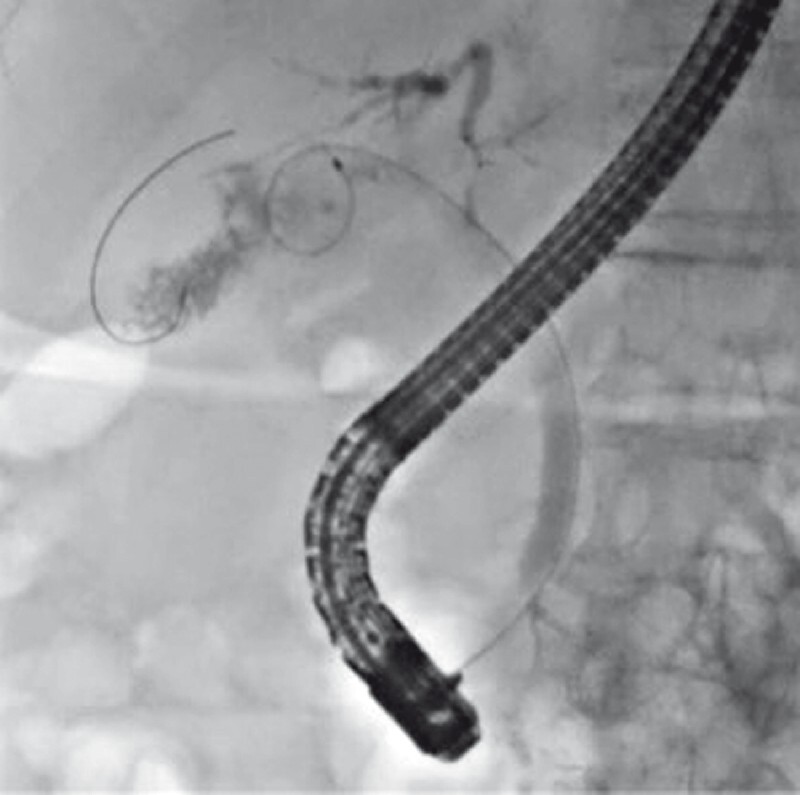
Cholangiography showing a filling defect with a characteristic appearance of a tumor.

**Fig. 3:**
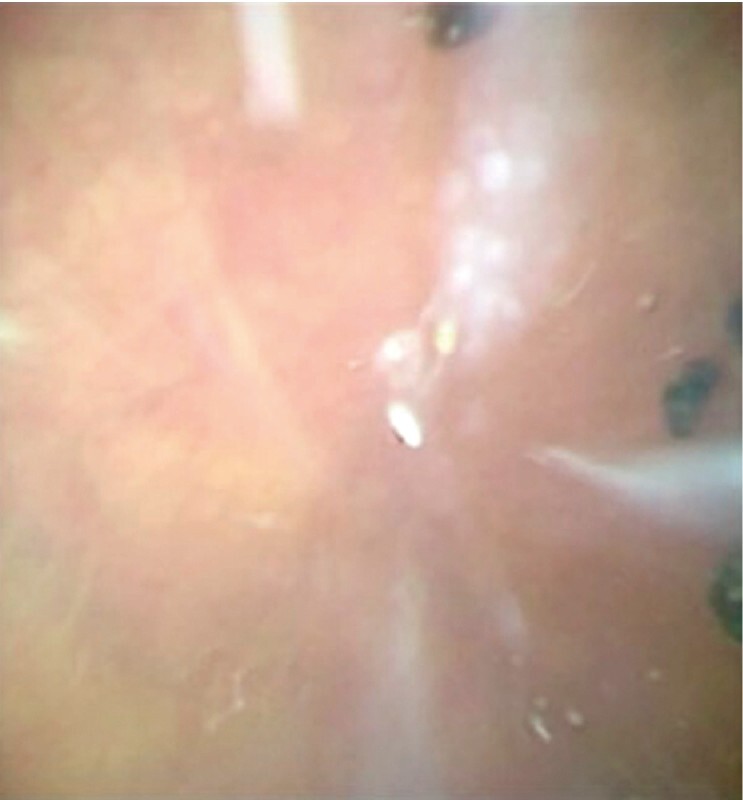
Visualization of the gallbladder from the novel cholangioscope.

**Fig. 4:**
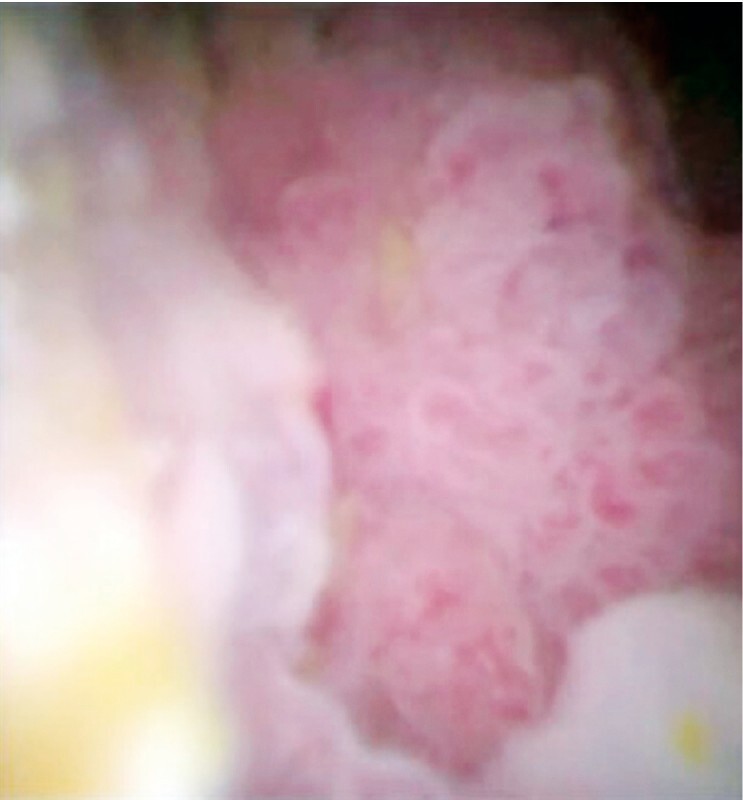
Identification of the gallbladder tumor on cholangioscopy.

**Fig. 5:**
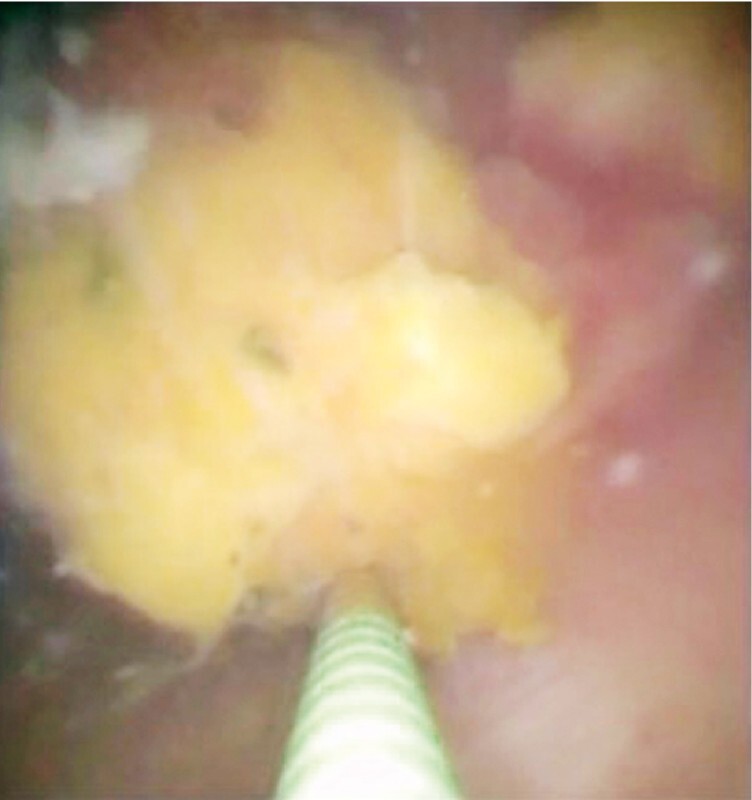
Tumor biopsy was performed successfully.

**Video 1** Forceps biopsy of a gallbladder tumor performed under cholangioscopy guidance using a novel cholangioscope.


In conclusion, the tapered shape of the novel eyeMAX cholangioscope makes it a viable option for performing transpapillary biopsy to obtain a histological diagnosis of gallbladder lesions.

Endoscopy_UCTN_Code_TTT_1AR_2AD

## Notes

Endoscopy E-Videos

https://eref.thieme.de/e-videos
*E-Videos* is an open access online section of the journal *Endoscopy* , reporting on interesting cases and new techniques in gastroenterological endoscopy. All papers include a high-quality video and are published with a Creative Commons CC-BY license. Endoscopy E-Videos qualify for HINARI discounts and waivers and eligibility is automatically checked during the submission process. We grant 100% waivers to articles whose corresponding authors are based in Group A countries and 50% waivers to those who are based in Group B countries as classified by Research4Life (see: https://www.research4life.org/access/eligibility/ ). This section has its own submission website at https://mc.manuscriptcentral.com/e-videos
